# Deep Learning-Based Detection of Separated Root Canal Instruments in Panoramic Radiographs Using a U^2^-Net Architecture

**DOI:** 10.3390/diagnostics15141744

**Published:** 2025-07-09

**Authors:** Nildem İnönü, Umut Aksoy, Dilan Kırmızı, Seçil Aksoy, Nurullah Akkaya, Kaan Orhan

**Affiliations:** 1 Department of Endodontics, Faculty of Dentistry, Near East University, 99138 Mersin, Turkey; umut.aksoy@neu.edu.tr (U.A.); dilan.kirmizi@neu.edu.tr (D.K.); 2 Department of Dentomaxillofacial Radiology, Faculty of Dentistry, Near East University, 99138 Mersin, Turkey; secil.aksoy@neu.edu.tr; 3 Dentmetria Inc., 34726 İstanbul, Turkey; nurullah@nakkaya.com; 4 Department of Dentomaxillofacial Radiology, Faculty of Dentistry, Ankara University, 06560 Ankara, Turkey; call53@yahoo.com; 5 Medical Design Application and Research Center (MEDITAM), Ankara University, 06560 Ankara, Turkey

**Keywords:** separated endodontic instruments, artificial intelligence, deep learning, panoramic radiographs, U^2^-Net

## Abstract

**Background:** Separated endodontic instruments are a significant complication in root canal treatment, affecting disinfection and long-term prognosis. Their detection on panoramic radiographs is challenging, particularly in complex anatomy or for less experienced clinicians. **Objectives:** This study aimed to develop and evaluate a deep learning model using the U^2^-Net architecture for automated detection and segmentation of separated instruments in panoramic radiographs from multiple imaging systems. **Methods:** A total of 36,800 panoramic radiographs were retrospectively reviewed, and 191 met strict inclusion criteria. Separated instruments were manually segmented using the Computer Vision Annotation Tool. The U^2^-Net model was trained and evaluated using standard performance metrics: Dice coefficient, IoU, precision, recall, and F1 score. **Results:** The model achieved a Dice coefficient of 0.849 (95% CI: 0.840–0.857) and IoU of 0.790 (95% CI: 0.781–0.799). Precision was 0.877 (95% CI: 0.869–0.884), recall was 0.847 (95% CI: 0.839–0.855), and the F1-score was 0.861 (95% CI: 0.853–0.869). **Conclusions:** These results demonstrate a strong overlap between predictions and ground truth, indicating high segmentation accuracy. The U^2^-Net model showed robust performance across radiographs from various systems, suggesting its clinical utility in aiding detection and treatment planning. Further multicenter studies are recommended to confirm generalizability.

## 1. Introduction

Root canal treatment (RCT) is a widely performed endodontic procedure aimed at preserving teeth affected by pulpal inflammation, infection, trauma, etc. The procedure involves removing infected or necrotic pulp tissue, disinfecting the root canal system, followed by obturation using biocompatible materials to restore tooth functionality and prevent microbial invasion [1]. The success of RCT depends heavily on thorough root canal cleaning and shaping, primarily achieved using nickel–titanium (NiTi) or stainless steel instruments [2]. However, instrument fracture is a common complication that can obstruct canal disinfection, hinder obturation, and compromise long-term treatment outcomes. Separated instruments can act as potential niduses for bacterial contamination, increasing the risk of periapical pathology and treatment failure [3]. Early detection and proper management are crucial for ensuring an optimal endodontic prognosis [4].

Instrument separation can occur at any stage of endodontic treatment, with an incidence ranging from 0.7% to 6.0% per canal or tooth [3]. Clinical studies indicate that mandibular molars are most commonly affected, especially in the apical third of the root, where retrieval is most challenging [5]. Accurate diagnosis of fractured instruments is crucial for treatment planning, guiding the decision to leave, bypass, or remove the fragment [6].

Clinicians use various methods to detect separated instruments during root canal treatment, primarily relying on clinical examination and radiographic imaging [6]. In some cases, a fractured instrument can be directly visualized in the coronal portion of the canal or detected through tactile feedback during instrumentation. However, when separation occurs in deeper or less accessible areas, radiographic imaging becomes essential for precise localization [7].

Periapical radiographs, orthopantomograms (OPGs), and cone beam computed tomography (CBCT) are commonly used for this purpose. While CBCT provides three-dimensional visualization and improves diagnostic accuracy, its reliability can be affected by artifacts from metallic objects such as canal posts and separated instruments, as well as radiopaque materials such as gutta-percha [8]. Additionally, CBCT exposes patients to higher radiation doses compared to conventional radiography [9]. OPGs and periapical radiographs are widely used due to their lower radiation exposure and accessibility; however, they have limitations in detecting small, radiopaque objects such as instrument fragments, especially when overlapping structures obscure visualization [8].

Artificial intelligence (AI) is transforming diagnostic imaging by enabling automated, rapid, and accurate detection of dental pathologies [10,11]. Machine learning, particularly deep learning (DL) methods, has shown significant potential in detecting periapical lesions, root fractures, and anatomical variations [12]. Convolutional neural networks (CNNs) have been widely applied in endodontic diagnostics, demonstrating promising results in radiographic image analysis [11,13]. However, studies focusing on AI-based detection of separated endodontic instruments remain limited [14,15,16,17]. Among these, only one study has been conducted using panoramic radiographs [16], while the others have relied on periapical images. Given the radiographic challenges in identifying instrument fragments due to their similarity to root filling materials, a more advanced segmentation-based approach may enhance detection accuracy. The U^2^-Net deep learning model, initially developed for salient object detection, has demonstrated strong performance in medical image segmentation [18,19]. In this study, we propose utilizing U^2^-Net for the automatic detection and segmentation of separated root canal instruments in orthopantomograms (OPGs). The primary aim is to evaluate its effectiveness in distinguishing instrument fragments from surrounding structures, reducing diagnostic variability, and enhancing clinical decision-making. To the best of our knowledge, this is the first study to apply U^2^-Net for this purpose, potentially providing new insights into AI-based endodontic diagnostics.

## 2. Materials and Methods

This retrospective study utilized 36,800 OPGs acquired from three different devices. After applying the inclusion and exclusion criteria (detailed in Table 1), the eligible OPGs were imported into the Computer Vision Annotation Tool (CVAT) for semantic segmentation of separated endodontic instruments (SEIs).

### 2.1. Data Preparation and Labelling

Out of 36,800 OPGs, 191 were used for this study. All OPGs were acquired using the Orthophos SL 3D (Dentsply Sirona, Bensheim, Germany) with parameters of 60–90 kVp and 3–16 mA, Orthophos XG (Dentsply Sirona, Bensheim, Germany) at 60–90 kVp and 3–16 mA, and PM 2002 CC Proline (Planmeca, Helsinki, Finland) at 60–70 kVp and 2–7 mA.

The OPGs, originally in DICOM format, were converted to PNG and uploaded to the CVAT for annotation. Manual pixel-wise segmentation was performed using CVAT’s polygon-based tools, which allowed precise delineation of SEI boundaries.

To ensure diagnostic accuracy and consistency, each image was independently evaluated and annotated by two experienced endodontists. SEIs were distinguished from other radiopaque structures—such as gutta-percha or metal posts—based on a combination of morphological features (e.g., shape, radiodensity, position within the canal) and radiographic confirmation.

To assess annotation reliability, inter-rater agreement was calculated using Cohen’s kappa (κ) statistic across all 191 annotated cases. The resulting κ value of 0.95 indicated almost perfect agreement.

Any discrepancies between the two annotators were reviewed and resolved by a dentomaxillofacial radiologist. The final annotation set reflects these reviewed and validated ground truth segmentations.

### 2.2. Model Pipeline

The pipeline in this study consisted of several key steps. Initially, the images were preprocessed to improve quality. Subsequently, a pixel-wise classification was applied to distinguish separated endodontic instruments from the background. Finally, the segmented instruments were extracted for further analysis, ensuring accurate identification of the items of interest.

### 2.3. Preprocessing

In the current study, minimal preprocessing was applied to preserve the native pixel intensity distribution of the panoramic radiographs. A simple per-image min–max normalization technique was used, in which the minimum pixel intensity of each image was subtracted and the result divided by the maximum pixel value of the same image.

To explore the potential benefits of more advanced preprocessing, Contrast Limited Adaptive Histogram Equalization (CLAHE) was applied. Although CLAHE is designed to enhance local contrast, its implementation in this context resulted in over-enhancement of radiopaque regions and diminished clarity of subtle anatomical boundaries. Consequently, segmentation performance decreased, with the U^2^-Net model achieving a Dice score of 0.827 following CLAHE, compared to 0.849 when using min–max normalization alone.

Standard data augmentation techniques—such as random flipping, rotation, and brightness adjustment—were evaluated. However, due to the fixed anatomical orientation of panoramic radiographs, only horizontal flipping was considered clinically appropriate. This transformation, although feasible, did not result in any measurable improvement in validation or test performance. Therefore, data augmentation was not included in the final model.

The final dataset was randomly divided into training (60%), validation (20%), and testing (20%) subsets.

### 2.4. Semantic Segmentation

Semantic segmentation was conducted to classify each pixel within an image as either background or representing a separated root canal instrument. To identify the optimal configuration, a systematic evaluation of model architectures and loss functions was performed. All models were trained on the same dataset to ensure comparability. Performance was primarily assessed using the Dice similarity coefficient, and the model yielding the highest score was selected for further analysis. Comprehensive results are summarized in Table 2.

**Figure 1:**
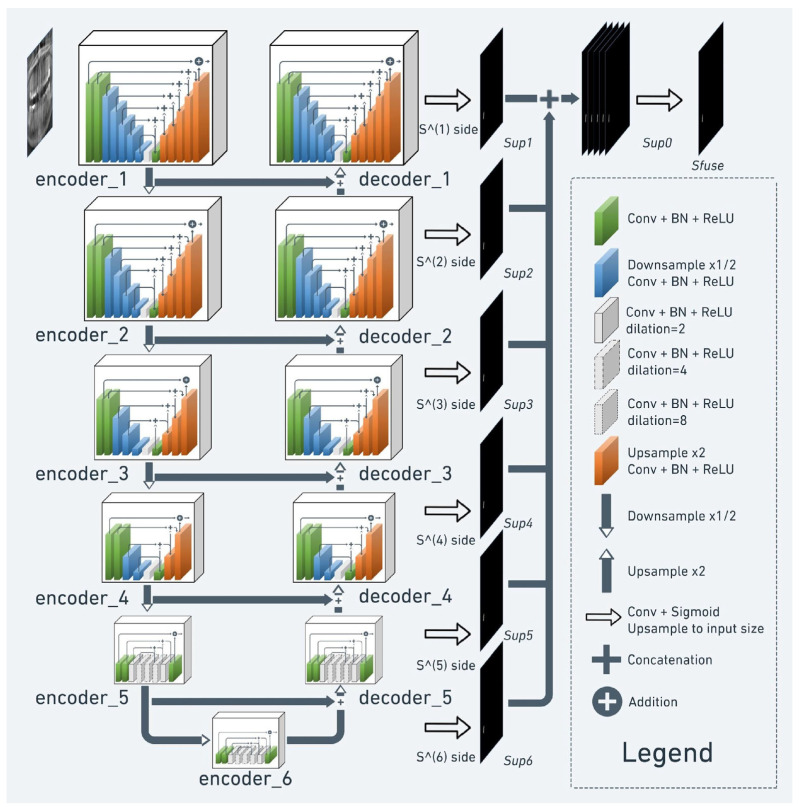
U^2^-Net architecture.

The U^2^-Net architecture—a refined variant of the traditional U-Net—was chosen. This model follows an encoder–decoder framework: the encoder (down convolution) captures semantic features, while the decoder (up sampling) reconstructs spatial detail. This structure enables the network to retain both semantic depth and spatial precision, which are essential for accurate pixel-level classification (Figure 1).

### 2.5. Implementation

The proposed deep learning algorithm was implemented using the U^2^-Net model with end-to-end training and evaluation. All experiments were conducted using the publicly available Python-based (Version 3.12) U^2^-Net implementation and carried out on an NVIDIA^®^ GeForce^®^ RTX 2080 Ti GPU. The model architecture was designed to accept input images of size 512 × 1024 with a single channel, classifying each pixel into one of two possible classes.

Model training was optimized using the AdamW optimizer with a learning rate of 0.0002. To address class imbalance during segmentation, a weighted sparse categorical Dice loss function was employed. In our implementation, the weighted Dice loss was computed by first determining the class weights based on the frequency of each class in the training dataset. These weights were inversely proportional to the class frequencies, thereby giving higher importance to underrepresented classes.

The Dice coefficient was calculated for each class individually by measuring the intersection between the predicted and ground truth masks, divided by their combined sum. Each class’s Dice score is the sum of these weighted class scores divided by the sum of weights. The final weighted Dice coefficient was obtained by summing these weighted class scores divided by the sum of weights. Consequently, the weighted Dice loss was defined as one minus this weighted coefficient, ensuring that minimizing the loss maximized the similarity between the predictions and the ground truth while effectively addressing class imbalance.

The model was trained for 500 epochs with a batch size of 4, and the version achieving the highest validation Dice score was selected for final evaluation.

## 3. Results

The U^2^-Net model demonstrated high accuracy in segmenting separated root canal instruments on the test dataset. The Dice coefficient, a key metric for evaluating segmentation performance, was 0.849 (95% CI: 0.840–0.857), indicating a strong overlap between the predicted and ground truth segmentations. Similarly, the IoU value was 0.790 (95% CI: 0.781–0.799), further confirming the model’s ability to accurately delineate the instrument fragments within radiographic images.

**Figure 2:**
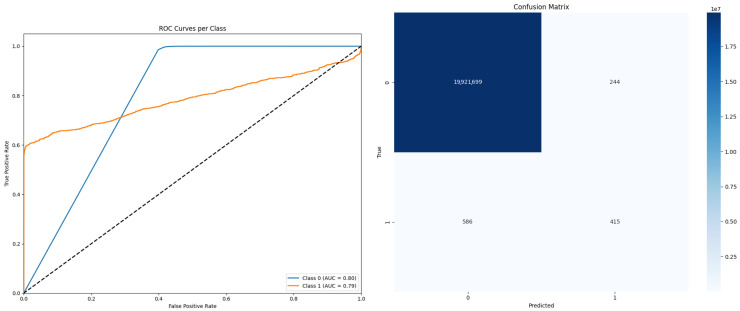
ROC curves and confusion matrix for model performance. The left panel shows the ROC curves for Class 0 (AUC = 0.80) and Class 1 (AUC = 0.79), demonstrating strong model performance. The confusion matrix shows high accuracy with minimal false positives and false negatives, indicating overall robust classification accuracy.

In addition to segmentation accuracy, the precision of the model was 0.877 (95% CI: 0.869–0.884), signifying a high proportion of correctly identified instrument fragments relative to the total predicted positives. Recall, which quantifies the model’s sensitivity in identifying all true positive cases, was 0.847 (95% CI: 0.839–0.855). The balance achieved between precision and recall is reflected in the F1 score, which reached 0.861 (95% CI: 0.853–0.869), demonstrating the effectiveness of the model in reducing both incorrect positive and negative predictions (Figure 2).

**Figure 3:**
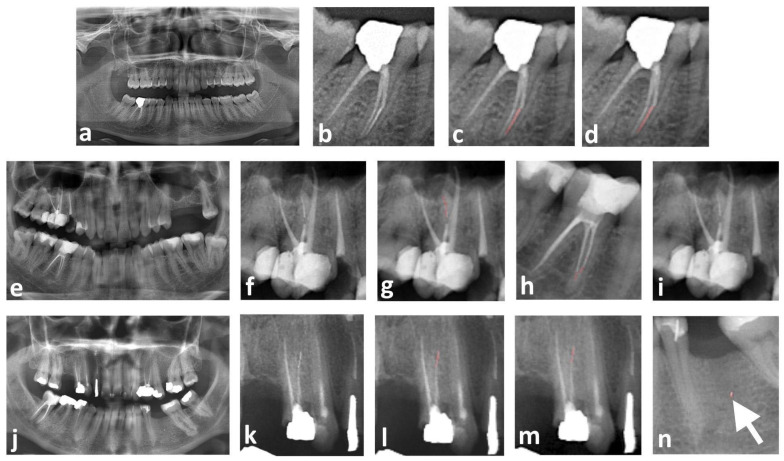
Representative examples demonstrating the segmentation performance of the proposed U^2^-Net-based deep learning model for detecting SEIs in panoramic radiographs. (**a**–**d**) True positive case: (**a**) original unannotated panoramic radiograph used as input, (**b**) cropped region highlighting the tooth with the SEI, (**c**) expert-drawn ground truth mask of the SEI, (**d**) AI model prediction, showing precise segmentation matching the ground truth. (**e**–**i**) False negative case: (**e**) original unannotated panoramic radiograph, (**f**) cropped view of the region with the SEI, (**g**) expert-drawn ground truth mask indicating the SEI, (**h**) AI misidentifies a root canal filling material in a different tooth as an SEI, (**i**) AI fails to detect the actual SEI. (**j**–**n**) Partial false positive case: (**j**) original unannotated panoramic radiograph, (**k**) cropped region containing the SEI, (**l**) expert-drawn ground truth mask of the SEI, (**m**) correct identification of the SEI by the AI, (**n**) AI also incorrectly segments an amalgam particle in an extraction socket as an additional SEI (white arrow). (Red color in the figure indicates the segmented regions of SEIs, either drawn by the expert or predicted by the AI model.)

These results indicate that the U^2^-Net model provides robust and reliable segmentation performance, with a low false-negative rate, making it a promising tool for detecting separated instruments in panoramic radiographs (Figure 3). The strong alignment between segmented regions and ground truth labels highlights the model’s potential clinical applicability, ensuring more accurate and efficient identification of instrument fractures in routine dental practice.

**Figure 4:**
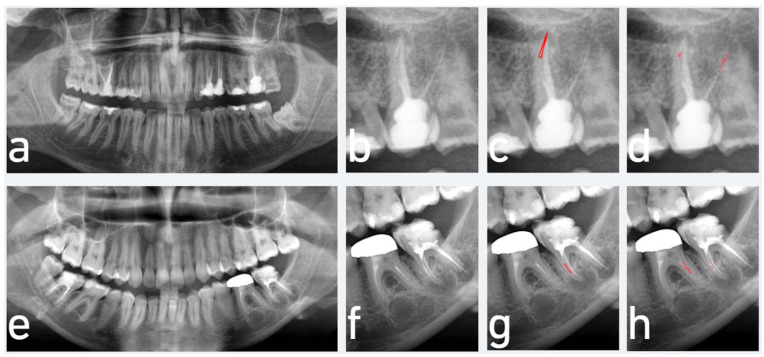
Segmentation errors in cases involving overlapping anatomical structures. (**a**,**e**) Input panoramic radiographs; (**b**,**f**) cropped images focusing on teeth with separated endodontic instruments (SEIs); (**c**,**g**) ground truth segmentations annotated by experts; (**d**,**h**) AI-generated segmentations, showing only partial detection of the SEIs. Additionally, the model misidentified adjacent RCT materials—such as in the distobuccal canal in (**d**) and in the distal root canal of tooth #36 in (**h**)—as SEIs. These errors resulted from anatomical superimposition, which interfered with accurate SEI localization. (Red color in the figure indicates the segmented regions of SEIs, either drawn by the expert or predicted by the AI model.)

Systematic error analysis revealed a systematic error index of 0.053, indicating a slight tendency of the AI model to underpredict the presence of SEIs compared to the expert annotator. This reflects a modest directional bias toward conservatism in the model’s predictions. A qualitative evaluation of the model’s performance on the test set revealed recurring misclassification patterns associated with anatomical superimposition. In the maxilla, errors were observed in areas where the maxillary sinus or adjacent roots overlapped the root canals, obscuring the visibility of separated instruments. In the mandible, detection failures were predominantly noted in molar teeth with two mesial canals, where canal overlap in radiographic projections made accurate localization of the fractured instruments challenging (Figure 4). These findings suggest that the model struggles particularly with complex anatomical overlap, which compromises the visibility of key radiographic features required for correct segmentation.

**Figure 5:**
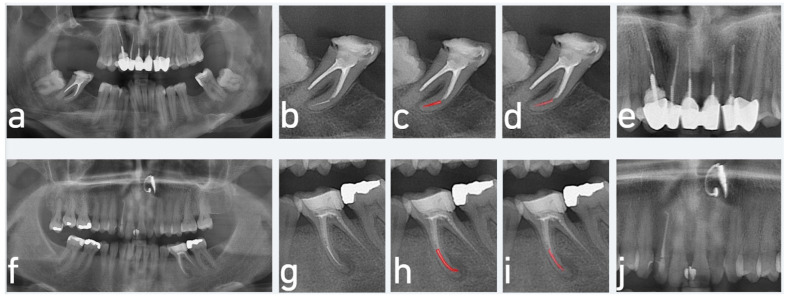
Representative examples demonstrating successful SEI detection and the model’s robustness against imaging artifacts. (**a**,**f**) Input panoramic radiographs; (**b**,**g**) cropped views of teeth with SEIs; (**c**,**h**) ground truth segmentations annotated by the expert; (**d**,**i**) AI-generated segmentations accurately identifying the SEIs; (**e**) maxillary teeth with metal posts; the model correctly did not misclassify them as SEIs; (**j**) a case with overextended root filling material in tooth #12 and a piercing artifact; the model correctly differentiated these radiopaque structures from SEIs. (Red color in the figure indicates the segmented regions of SEIs, either drawn by the expert or predicted by the AI model.)

Notably, several cases in the test set included teeth with intracanal metallic posts, dentin pins, or radiopaque artifacts. Despite these potential confounding features, the model did not misidentify posts or pins as separated endodontic instruments and was able to correctly segment the true instrument fragments in such cases (Figure 5). This highlights the model’s robustness and its ability to distinguish between diagnostically similar radiopaque structures.

## 4. Discussion

The U-Net architecture has been widely applied in dentistry for various tasks, including tooth segmentation, caries detection, restoration and root canal filling identification, crown–bridge evaluation, implant segmentation [20], third molar and mandibular canal relationship assessment [21], pharyngeal airway measurement [22], and periapical lesion detection [11,23].

U^2^-Net, a nested version of U-Net originally designed for salient object detection, introduces a two-level architecture that enables efficient multi-scale feature extraction while preserving high-resolution outputs. Its core component, the Residual U-block (RSU), allows the model to maintain spatial detail and depth without requiring pre-trained backbones, making it suitable for detecting small or low-contrast structures in complex radiographic evaluations [18]. The choice of U^2^-Net was guided by its superior ability to preserve fine-grained features through nested residual U-blocks, making it well suited for segmenting small objects, such as SEIs, in panoramic radiographs. Compared to standard U-Net or other encoder–decoder architectures, U^2^-Net offers enhanced multi-scale feature extraction without requiring a pre-trained backbone, which improves adaptability to diverse datasets and eliminates dependency on external training priors. In dentistry, U^2^-Net has been successfully applied to tasks such as periapical lesion detection [24] and tooth segmentation [25] on panoramic radiographs, as well as head and neck tumor segmentation on positron emission tomography (PET) and CT images [26]. These features make it a promising tool for detecting subtle structures, such as SEIs, which often exhibit radiodensities similar to surrounding root canal materials.

Separated endodontic instruments present a clinical challenge due to their potential to compromise canal disinfection, prolong treatment, and adversely affect long-term outcomes, particularly when associated with apical pathology [27,28]. Although their incidence is relatively low, fractures often occur without visible signs—especially with rotary NiTi files—and are typically caused by torsional or flexural fatigue. The clinical approach to managing SEIs remains highly case-specific, influenced by canal anatomy, fragment location, the presence of periapical lesions, and potential iatrogenic risks [4]. This highlights the importance of early and accurate detection, as timely intervention can reduce complications and improve prognosis.

In the present study, a deep learning model was developed using U^2^-Net to detect separated endodontic instruments on panoramic radiographs obtained from three different imaging systems under standardized exposure settings. Prior to model training, all images were resized to 512 × 1024 pixels using bilinear interpolation to ensure compatibility with the network architecture while preserving anatomical proportions. This resizing step helped maintain consistency across input data without introducing distortion artifacts that could affect segmentation performance. The model achieved high diagnostic accuracy and demonstrated strong robustness across devices, indicating its potential for integration into various clinical workflows. Unlike previous studies that primarily relied on periapical images or datasets acquired from a single device, this approach is among the first to validate an AI model using panoramic radiographs from multiple platforms, thereby enhancing its generalizability and minimizing bias introduced by uniform imaging conditions.

Although the localization of fractured instruments and subsequent treatment planning are critical steps influencing tooth prognosis, preventive strategies to avoid such complications are equally important. In this context, Thakur et al. developed a machine learning-based model to assess the health status of endodontic instruments during treatment by analyzing force signals. Their system aimed to predict instrument fatigue and potential fractures before occurrence, functioning as a real-time preventive tool. While their approach differs in scope from this study—which focuses on the post-operative detection of already separated instruments using panoramic radiographs—both studies highlight the expanding role of artificial intelligence across different stages of endodontic procedures. This underscores the potential for integrating multiple AI modalities into future clinical decision-support systems [29].

These findings align with previous studies demonstrating the ability of convolutional neural networks to detect SEIs on radiographic images. Özbay et al. successfully applied Mask R-CNN to periapical radiographs; however, their dataset was limited to images obtained from a single radiographic unit, potentially restricting model robustness in varied clinical settings [15]. Similarly, while Büyük et al. introduced a Gabor-filtered CNN model that achieved high accuracy using panoramic images, their method required a preprocessing step [16]. In contrast, our model operates directly on standard panoramic radiographs, facilitating real-time use in dental practice.

Recent studies have investigated deep learning-based SEI detection. Özbay et al. reported high detection performance using Mask R-CNN on periapical images (mAP 98.8%) [15]. Çatmabacak and Çetinkaya evaluated five CNN architectures on annotated periapical datasets, with DenseNet201 yielding the best results (accuracy 90.5%, AUC 0.90). While these studies demonstrate the effectiveness of DL in SEI detection, they rely solely on periapical radiographs [14]. In contrast, Büyük et al. employed CNN and LSTM models on panoramic images, achieving 84.4% accuracy with Gabor-filtered CNNs. With these findings, our study developed a U^2^-Net model trained on panoramic radiographs from three devices under standardized parameters, achieving high diagnostic performance and cross-device generalizability [16]. A study by Çetinkaya et al. (2025) compared the performance of YOLOv8 and Mask R-CNN in detecting fractured endodontic instruments on periapical radiographs, demonstrating high diagnostic accuracy comparable to that of experienced clinicians. Their findings emphasized the effectiveness of both real-time object detection and segmentation models in radiographic evaluation of SEIs. However, like other previous studies, their dataset was limited to periapical radiographs, which offer higher resolution but narrower anatomical coverage. In contrast, our study uniquely employs panoramic radiographs, which provide a broader anatomical view [17].

Building upon the strengths of previous CNN-based models, our approach focused on maintaining high accuracy while enhancing adaptability across different devices. This is particularly important in clinical settings with hardware variability, helping to bridge the gap between AI development and practical application. Clinically, accurate SEI detection is essential, as the location and visibility of the fragment significantly influence treatment decisions and prognosis. Terauchi et al. emphasized that fragments in the coronal or middle third—and those visible on imaging—are more amenable to retrieval using minimally invasive techniques. In contrast, apical fragments, especially those not visible, are harder to manage and may require conservative strategies such as bypassing or retention [30]. Our model’s ability to detect such fragments across different panoramic systems suggests potential utility in supporting clinicians, particularly in anatomically complex or resource-limited settings.

Despite these promising results, this study has some limitations. Although three different radiographic devices were used, the dataset was derived from a single institution, which may affect external validity. Cases with severe artifacts were excluded to minimize noise during model training, which may still limit applicability in certain complex scenarios. Furthermore, k-fold cross-validation—a strategy commonly used to assess model generalizability in small datasets—was not implemented due to the extensive training time required by the segmentation model. Future studies with greater computational capacity may consider this approach to enhance robustness and reproducibility.

Future studies should explore multicenter datasets encompassing a broader range of anatomies and radiographic protocols. Combining periapical and panoramic radiographs may enhance sensitivity, particularly in apical regions with overlapping structures. Moreover, hybrid models integrating clinical data—such as tooth type, curvature, and treatment history—could improve predictive performance by adding contextual awareness to image-based analysis.

## 5. Conclusions

Overall, this study underscores the potential of deep learning technologies to enhance diagnostic accuracy in endodontics. By demonstrating the reliable detection of fractured instruments across panoramic radiographs from multiple devices, this study contributes to the integration of AI into daily clinical practice. With further refinement, such systems may evolve into real-time diagnostic aids, educational tools, and decision-support platforms, ultimately improving the safety and predictability of endodontic treatments.

## Figures and Tables

**Table 1:** The inclusion and exclusion criteria for the study.

Inclusion Criteria	Exclusion Criteria
Panoramic radiographs of single or multirooted RCT teeth with the presence of a radiographically confirmed separated endodontic instrument	Radiographs without the RCT and separated endodontic instruments
Patient with permanent teeth	Patient with primary teeth or without any teeth (edentulous patient)
Panoramic radiographs obtained using Orthophos SL 3D, Orthophos XG, and PM 2002 CC Proline, with standardized exposure settings (60–90 kV, 3–16 mA for Orthophos devices; 60–70 kV, 2–7 mA for Planmeca) to ensure consistency across imaging systems.	Radiographs taken with devices other than Orthophos SL 3D, Orthophos XG, or PM 2002 CC Proline, or with non-standard exposure settings, leading to variations in image quality.
Radiographs free of imaging artifacts such as motion blur, positioning errors, or foreign objects interfering with assessment.	Radiographs with significant imaging artifacts (motion blur, positioning errors) that compromise accurate evaluation.
Radiographs of teeth with complete root formation and no history of previous endodontic surgery.	Radiographs of teeth with evidence of previous endodontic surgery, retreatment, or root resorption affecting the periapical area.
Radiographs obtained with proper angulation and minimal distortion, ensuring accurate representation of the root canal anatomy and separated instruments.	Radiographs with severe distortion or non-standard angulation, misrepresenting the actual location of separated instruments.
No presence of large periapical lesions (>5 mm) that could interfere with the assessment of separated instruments.	Radiographs showing extensive periapical pathology or overlapping anatomical structures, making identification of separated instruments difficult.
Radiographs with RCT cases containing intracanal posts, pins, or other restorative materials	

**Table 2:** Comparison of different model architectures and loss functions trained on the same dataset.

Architecture	Cross-Entropy	Weighted CE	Dice	Weighted Dice
HRNet	0.618	0.642	0.673	0.672
Attention U-Net	0.659	0.751	0.782	0.803
ResUNet	0.651	0.689	0.665	0.661
U^2^-Net	0.6	0.696	0.847	0.863
UNet	0.652	0.775	0.81	0.774

## Data Availability

The data presented in this study are available on request from the corresponding author.

## Abbreviations

The following abbreviations are used in this manuscript:

RCT	Root canal treatment
NiTi	Nickel–titanium
OPGs	Orthopantomograms
CBCT	Cone beam computed tomography
AI	Artificial intelligence
DL	Deep learning
CNN	Convolutional neural networks
CVAT	Computer Vision Annotation Tool
IoU	Intersection over Union
LSTM	Long short-term memory
RSU	Residual u-block
